# Factors Influencing Evaluation Completion During Emergency Medicine Sub-internships: A Multi-site Observational Study

**DOI:** 10.7759/cureus.95750

**Published:** 2025-10-30

**Authors:** Brian Merritt, Ana S Tuttle, Julia Ruggieri, Rowan H Kelner, Robert Stephen, Patrick G Hughes, Allison M Beaulieu

**Affiliations:** 1 Emergency Medicine, The University of Utah, Salt Lake City, USA

**Keywords:** academic vs. community, emergency medicine, evaluation, gender, sub-internship

## Abstract

Introduction: Clinical rotation assessments are essential for evaluating student competencies, guiding feedback, and determining readiness for residency. Equitable sub-internship evaluations rely on complete and representative input from supervising attending physicians and senior residents. However, it remains unclear whether evaluation completion varies based on evaluator or student characteristics. This study examined completion rates of on-shift evaluations during emergency medicine (EM) sub-internships at two affiliated academic sites and assessed whether evaluator and student factors were associated with differences in evaluation completion.

Methods: A retrospective observational study was conducted across two level 1 trauma centers affiliated with a single EM residency program during the 2023-2024 academic year. All sub-internship students (n = 56) were included. After each shift, students invited supervisors to complete an online evaluation. Variables included evaluator gender, professional rank, clinical sites, and student gender. The primary outcome was evaluation completion rate. Descriptive statistics and 95% confidence intervals (Wilson method) were reported. Group differences were analyzed using Pearson’s chi-square and Fisher’s exact tests.

Results: A total of 56 students distributed 1,038 evaluation invitations, of which 658 (63.4%) were completed. Completion rates differed significantly by evaluator type, with residents completing 187 (74.5%) of 251 evaluations and advanced practice providers completing 10 (55.6%) of 18 evaluations (χ² = 21.65, p = 0.003). Evaluator gender was also associated with response rate: female evaluators completed 272 (67.5%) of 403 evaluations, compared to male evaluators who completed 386 (60.8%) of 635 (χ² = 4.49, p = 0.034). Completion rates were higher at the university-based site, with 392 (67.8%) of 578 evaluations completed, compared to 266 (57.8%) of 460 at the community-based site (χ² = 10.60, p = 0.001). No significant differences were observed by student gender: 262 (64.5%) of 406 evaluations were completed for women, and 393 (62.7%) of 632 for men (χ² = 0.30, p = 0.585).

Conclusion: Evaluation completion during EM sub-internships varied by evaluator type, evaluator gender, rotation site, student degree type, and rotation month, but not by student gender or specialty interest. These findings identify practical targets for improving equitable and representative evaluation practices. Generalizability is limited by single-program design, and multi-institutional studies are warranted to further explore these trends.

## Introduction

Evaluation and assessment of medical students during clinical rotations are essential for assessing students' competencies, providing actionable feedback, and determining readiness for residency [1]. These evaluations offer a structured mechanism to assess students' clinical skills, reasoning, medical knowledge, patient and team-centered communication, and professionalism [2]. Evaluations are typically completed by a range of healthcare professionals, including physicians, nurses, and other members of the healthcare team. These evaluations provide a comprehensive view of the student's performance from multiple perspectives. It is recommended that in emergency medicine (EM) clerkships, faculty and senior EM residents should be the primary evaluators of medical students during their EM rotation [2]. Evaluation data are also used to determine final rotation grades and are translated into the standardized letter of evaluation (SLOE) in EM [3]. The SLOE is a key tool that offers a comparative performance assessment that is essential for residency applications.

The SLOE is an important component of a medical student’s application to EM and can determine whether or not a student is offered an interview and where they rank on a residency program's list [4-6]. Given the significance of the SLOE in medical students’ applications to EM residency, it is crucial to examine the input data. On-shift assessments provide critical insight into the scoring and ranking process that contributes to the SLOE. These assessments help ensure that students are meeting the necessary standards and are prepared for the demands of their chosen specialty.

Understanding how different clinical environments influence student performance can help improve the quality and quantity of feedback in medical education and ensure that students receive the best possible training. This analysis could lead to more targeted improvements in clinical education programs, ultimately benefiting both students and patients. The purpose of this study is to evaluate whether faculty, student, and clinical site characteristics are associated with differences in the completion of medical student evaluations.

## Materials and methods

Study design and setting

This retrospective observational study was conducted at a single EM residency program in the United States during the 2023-2024 academic year. Medical student evaluation data were collected exclusively from quick-response (QR) code-based evaluations at two rotation sites: a tertiary care, university-based level 1 trauma center and a tertiary care, community-based level 1 trauma center.

Selection of participants

Eligible participants included fourth-year medical students from US allopathic and osteopathic medical schools who completed a four-week EM sub-internship (14 shifts in total, seven at each site) at both participating emergency departments during the 2023-2024 academic year. The inclusion criteria required that students complete at least one clinical shift during the study period and receive a QR code-based evaluation from their supervisor after the completion of their shift. Students were excluded if they did not complete any clinical shifts, rotated at non-participating sites, did not receive a QR code-based evaluation, or were not fourth-year medical students.

Interventions

At the conclusion of each shift, students provided a business card containing a QR code to their supervisor (attending, fellow, resident, or advanced practice provider). Scanning the QR code directed the evaluator to an online Qualtrics evaluation form, which included student names and pictures to ensure the correct student was matched to the appropriate evaluation. Students filled out a separate evaluation form at each shift, with the supervisor's name and shift information, which allowed tracking of distribution rates.

Measurements

Evaluations included both student- and evaluator-level information. Student variables included gender. Evaluator variables included clinical site, gender, and professional type. The number of evaluation cards distributed and the number submitted were recorded to determine response rates. All evaluation questions and a supervisory signature must be completed prior to submitting the form.

Outcomes

The primary outcome was the evaluation response rate, defined as the proportion of distributed evaluations that were completed, stratified by student and evaluator characteristics.

Analysis

Analyses were performed in R version 4.4.2 (R Foundation for Statistical Computing, Vienna, Austria) using the Tidyverse (v2.0.0), Janitor (v2.2.0), Broom (v1.0.5), and Binom (v1.1-1) packages. Descriptive statistics summarized categorical variables as counts and percentages. Response rates were reported with Wilson 95% confidence intervals. Associations between categorical variables and response rates were evaluated using Pearson’s chi-squared tests; Fisher’s exact test was used when expected cell counts were <5. Incomplete evaluations were not included in the data analysis.

## Results

A total of 57 students completed the EM sub-internship during the 2023-2024 academic year. Of the 1,038 evaluation invitations distributed to supervisors across both sites, 658 (63.4%) were completed. Student and evaluator characteristics are summarized in Table 1.

**Table 1:** Student and evaluator characteristics. Student counts/percentages reflect unique students (n = 56). Evaluator counts/percentages reflect evaluation invitations (n = 1,038). MD: doctor of medicine; DO: doctor of osteopathic medicine; EM: emergency medicine; NEM: non-emergency medicine.

Group	Characteristic	Category	n (%)
Students	Degree type	MD	36 (63.2%)
	Degree type	DO	21 (36.8%)
	Gender	Male	35 (61.4%)
	Gender	Female	22 (38.6%)
	Intended specialty	EM	45 (78.9%)
	Intended specialty	NEM	12 (21.1%)
Evaluators	Clinical site	Academic	578 (55.7%)
	Clinical site	Community	460 (44.3%)
	Gender	Male	635 (61.2%)
	Gender	Female	403 (38.8%)
	Rank	Community faculty	461 (44.4%)
	Rank	Resident	251 (24.2%)
	Rank	Assistant professor	113 (10.9%)
	Rank	Professor	68 (6.6%)
	Rank	Associate professor	55 (5.3%)
	Rank	Visiting instructor	44 (4.2%)
	Rank	Clinical attending	28 (2.7%)
	Rank	Advanced practice providers	18 (1.7%)

Response rates by evaluator characteristics are presented in Table 2. Advanced practice providers completed 10 (55.6%) of 18 distributed evaluations, while residents completed 187 (74.5%) of 251 evaluations, representing the lowest and highest completion rates by evaluator type, respectively. There were statistically significant differences across evaluator types (χ² = 21.65, df = 7, p = 0.003) (Table 2).

**Table 2:** Response rates by evaluator type. Distributed (n), responses, and rate with Wilson 95% CI. Pearson's chi-squared test, χ² = 21.65, df = 7, p = 0.003.

Category	Distributed (n)	Responded (n)	No response (n)	Response rate	95% CI
Resident	251	187	64	74.5%	68.8%–79.5%
Professor	68	46	22	67.6%	55.8%–77.6%
Visiting instructor	44	29	15	65.9%	51.1%–78.1%
Clinical attending	28	18	10	64.3%	45.8%–79.3%
Associate professor	55	35	20	63.6%	50.4%–75.1%
Assistant professor	113	66	47	58.4%	49.2%–67.1%
Community attending	461	267	194	57.9%	53.4%–62.3%
Advanced practice provider	18	10	8	55.6%	33.7%–75.4%

Female evaluators had a higher response rate, completing 272 (67.5%) of 403 distributed evaluations, whereas male evaluators completed 386 (60.8%) of 635 evaluations (χ² = 4.49, df = 1, p = 0.034) (Table 3).

**Table 3:** Response rates by evaluator gender. Distributed (n), responses, and rate with Wilson 95% CI. Pearson's chi-squared test, χ² = 21.65, df = 7, p = 0.003.

Category	Distributed (n)	Responded (n)	No response (n)	Response rate	95% CI
Female	403	272	131	67.5%	62.8%–71.9%
Male	635	386	249	60.8%	56.9%–64.5%

Student gender was not significantly associated with evaluation response rate: 262 (64.5%) of 406 evaluations were completed for females, compared to 393 (62.7%) of 632 for males (χ² = 0.30, df = 1, p = 0.585) (Table 4).

**Table 4:** Response rates by student gender. Distributed (n), responses, and rate with Wilson 95% CI. Pearson's chi-squared test, χ² = 0.30, df = 1, p = 0.585.

Category	Distributed (n)	Responded (n)	No response (n)	Response rate	95% CI
Female	406	262	144	64.5%	59.8%–69.0%
Male	632	396	236	62.7%	58.8%–66.3%

Response rate also differed by clinical site, with higher rates at the university-based site (392 (67.8%) of 578) compared to the community-based site (266 (57.8%) of 460) (χ² = 10.60, df = 1, p = 0.001) (Table 5).

**Table 5:** Response rates by clinical location. Distributed (n), responses, and rate with Wilson 95% CI. Pearson's chi-squared test, χ² = 10.60, df = 1, p = 0.001.

Category	Distributed (n)	Responded (n)	No response (n)	Response rate	95% CI
Academic	578	392	186	67.8%	63.9%–71.5%
Community	460	266	194	57.8%	53.3%–62.3%

## Discussion

This retrospective study was performed to ascertain the impact of evaluator and site characteristics on medical student evaluations. The primary outcome was the completion rate of student evaluations, with evaluator characteristics assessed, including evaluator type, gender, and practice (academic versus community).

Evaluators at the academic site were significantly more likely to complete the student evaluation than those at the community site. Academic faculty are often incentivized to complete evaluations to receive additional support or financial compensation [7]. In addition, contact information for academic faculty was readily available, facilitating the direct distribution of reminders. In contrast, community faculty contact information was less accessible, and their compensation structure is generally not tied to evaluation compensation. However, they are motivated by the opportunity to influence their future workforce through their feedback.

Residents had the highest evaluation completion rates, likely due to their dual motivation to contribute to the development of their residency program and their ability to work across both clinical sites. Additionally, EM students often perceive residents as playing a more active role in their education compared to attending physicians [8,9]. This perception may have led students to distribute more evaluations to residents, contributing to the higher completion rates observed. In contrast, advanced practice providers had lower completion rates, which may be attributed to their limited interaction with students and residents, as well as the absence of financial or other incentives tied to evaluation completion.

Although students completed a similar number of shifts at both sites, fewer evaluations were distributed at the community site compared to the academic center, despite both having sufficient overall distribution. This likely contributed to the lower total number of completed evaluations and a reduced percent yield. However, the 10% difference in completion rates between sites is notable and suggests a true decrease in response rate at the community site. This discrepancy is likely due to differences in shift structure: at the academic center, students are consistently paired with a designated attending and resident team, whereas at the community site, students may staff patients with multiple attendings, making consistent evaluation more difficult.

Faculty evaluation completion rates did not appear to be influenced by student gender. However, female evaluators were more likely to complete evaluation forms than their male counterparts. While there is no existing literature directly linking gender to evaluation or feedback form completion rates, a prior study showed that female faculty often receive lower scores by trainees in domains related to autonomy [10]. One possible explanation is that increased oversight or engagement in educational activities may contribute to higher evaluation completion rates among female evaluators. Further research is needed to explore this trend and better understand the underlying factors influencing evaluator behavior.

This study has several limitations. First, it was conducted within a single EM residency program across two affiliated clinical sites, which may limit the generalizability of findings to other institutions, specialties, or geographic regions. Second, evaluation completion was voluntary and relied on student-initiated distribution of QR code evaluation cards, potentially introducing selection bias. Third, evaluator-specific factors such as prior teaching experience, workload, or familiarity with the evaluation system were not captured and may have influenced completion rates. Lastly, the study focused solely on evaluation completion rates and basic scoring metrics; it did not assess the quality or content of the evaluations themselves.

## Conclusions

Evaluation completion during emergency medicine sub-internships varied significantly by evaluator type, evaluator gender, and clinical site. These findings suggest that structural and contextual factors influence evaluation practices and may contribute to inequities in assessment data. Improving evaluator accessibility, enhancing faculty engagement, and standardizing the evaluation process may promote more equitable and representative student assessments. Future multi-institutional studies are needed to validate these findings and explore strategies to optimize evaluation completion and quality across diverse clinical settings.
